# Theoretical and NMR-based Conformational Analysis of Phosphodiester-linked Disaccharides

**DOI:** 10.1038/s41598-017-09055-x

**Published:** 2017-08-21

**Authors:** Alexey G. Gerbst, Andrei V. Nikolaev, Dmitry V. Yashunsky, Alexander S. Shashkov, Andrey S. Dmitrenok, Nikolay E. Nifantiev

**Affiliations:** 1 Laboratory of Glycoconjugate Chemistry, N.D. Zelinsky Institute of Organic Chemistry, Russian Academy of Sciences, Leninsky prospect 47, 119991 Moscow, Russian Federation; 2 University of Dundee, College of Life Sciences, Dundee, United Kingdom

## Abstract

The conformational behaviour of three phosphate-bridged dimannosides was studied by means of NMR and computational molecular modelling. First, the conformations of the phosphodiester linker were determined by quantum chemistry methods using dimethyl phosphate as a model. Then, a series of conformations was constructed for each of the studied molecules. Preliminary molecular dynamics (MD) simulations revealed that the inclusion of a cation had a drastic influence on the obtained results. Additionally, triethylammonium had the same effect as sodium as the counter-ion. After that, another series of MD simulations was run. The resulting MD trajectories were used to define the conformations responsible for the observed nuclear Overhauser effects and inter-nuclear coupling.

## Introduction

Phosphodiester bridges represent an important functional fragment widely encountered in natural polysaccharides including capsular bacterial polysaccharides (examples of producers: *Streptococcus pneumonia* ^1–9^, *Haemophilus influenzae* ^10–14^, *Escherichia coli* ^15–17^) and others (see review paper^18^). The polysaccharides bearing phosphodiester bridges (i.e., structurally phosphoglycans) are of ongoing interest as models for immunological investigations, vaccine design, diagnostics development and other purposes. For example, an exocapsular phosphomannan from *Pichia holstii* ^19^ was used as a raw material for the synthesis of anti-cancer drug-candidate PI-88^19^, which was produced by hydrolysis and partial sulfation of the phosphomannan.

**Figure 1:**
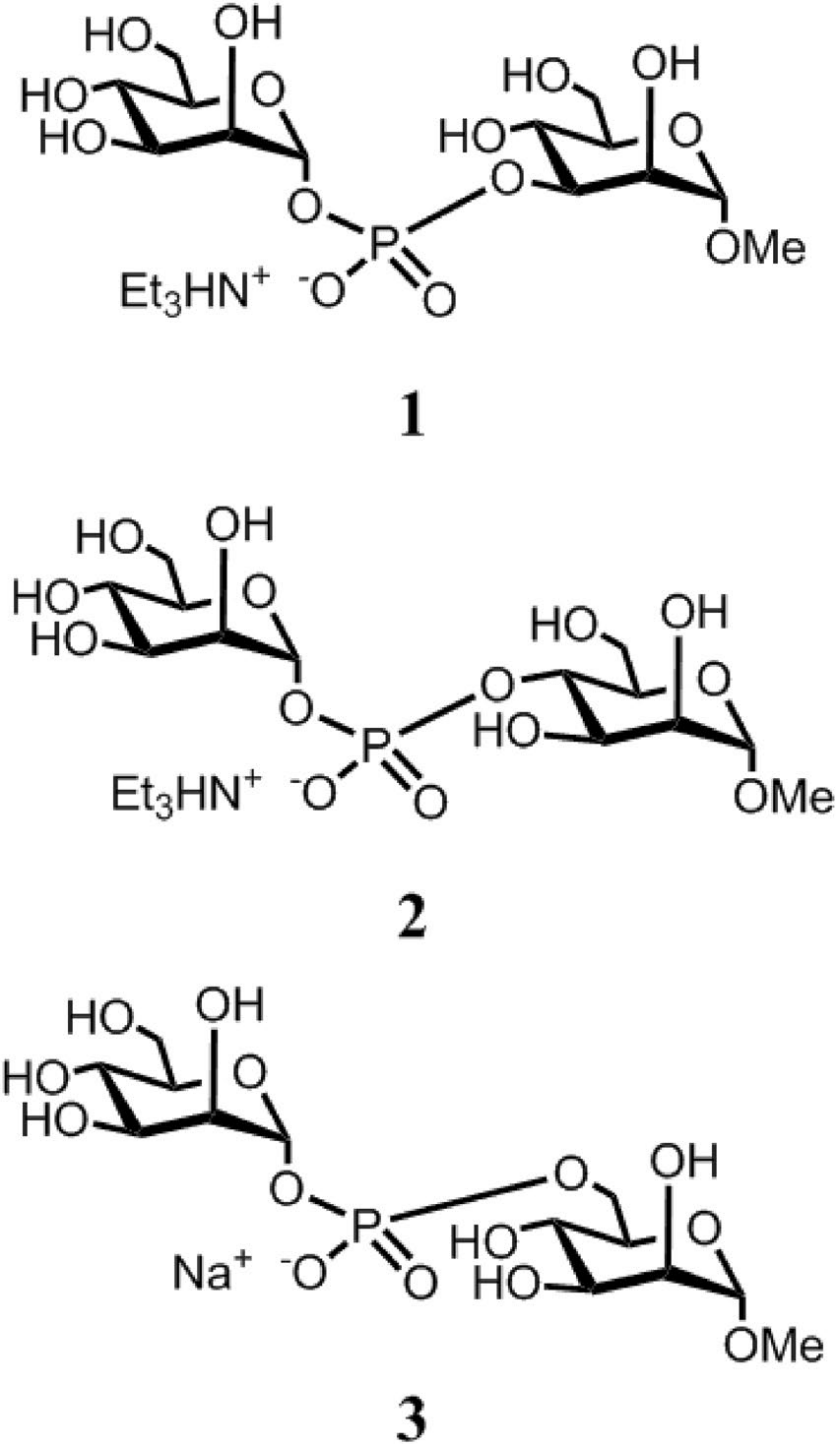
The structures of the studied phosphodimannosides.

Meanwhile, the conformational properties of oligosaccharide fragments around phosphodiester bridges have not been systematically studied until now. The studies to date are limited to model *ab initio* calculations of a tetrahydropyranyl phosphate derivative^20^ and investigation of the molecular dynamics of glucosyl nucleotides^21^. To fill this gap, we conduct systematic conformational studies of oligo- and polysaccharides bearing phosphodiester bridges. Herein, we report the results of theoretical (density functional theory (DFT) and molecular mechanics calculations) and NMR-based conformational studies of a series of three isomeric glycosyl phosphosaccharide compounds (Fig. 1) in which the α-D-mannosylphosphate unit is connected to the methyl α-D-mannoside section at O-3 (1), O-4 (2), and O-6 (3). Fragments related to compound 3 have been discovered in the chains of cell walls and extracellular phosphomannas of yeast^22–30^. Phosphodiesters **1** and **2** have been prepared as model compounds, since those types of intersaccharidic linkage [(1 → 3) and (1 → 4)] are common in natural bacterial polysaccharides^18^, but between different type of monosaccharides (not for α-D-Man*p*). The synthesis of compounds **1–3** was described previously^31, 32^. Their ^1^H and ^13^C NMR data are given in Tables 1 and 2. The assignment of the chemical shifts was performed by the use of 2D COSY, HSQC and HMBC techniques.

**Table 1:** ^1^H chemical shifts (δ, ppm; CD_3_OD) of compounds **1–3**.

Compound	Residue	H1	H2	H3	H4	H5	H6_a,b_
**1**	α-D-Man*p*-(1-*P*-	5.54	3.93	3.81	3.64	3.84	3.86, 3.75
	-3)-α-D-Man*p*-OMe	4.69	4.06	4.34	3.82	3.57	3.89, 3.72
**2**	α-D-Man*p*-(1-*P*-	5.54	3.96	3.82	3.68	3.82	3.87, 3.75
	-4)-α-D-Man*p*-OMe	4.68	3.84	3.88	4.30	3.57	3.85, 3.83
**3**	α-D-Man*p*-(1-*P*-	5.49	3.94	3.83	3.63	3.83	3.83, 3.71
	-6)-α-D-Man*p*-OMe	4.65	3.79	3.68	3.72	3.58	4.16, 4.12

**Table 2:** ^13^C chemical shifts (δ, ppm; CD_3_OD) of compounds **1–3**.

Compound	Residue	C1	C2	C3	C4	C5	C6
**1**	α-D-Manp-(1-P-	98.2	72.6	72.1	68.6	75.9	62.9
	-3)-α-D-Manp-OMe	102.6	71.2	78.8	67.9	74.6	63.1
**2**	α-D-Manp-(1-P-	98.5	72.5	72.1	68.5	75.9	63.0
	-4)-α-D-Manp-OMe	102.5	71.9	72.3	73.6	73.4	62.6
**3**	α-D-Manp-(1-P-	97.7	72.2	71.7	68.3	75.5	62.7
	-6)-α-D-Manp-OMe	102.5	71.8	72.2	68.0	73.2	66.0

## Results and Discussion

### Conformational analysis of the dimethyl phosphate as a model of the phosphodiester bridge

**Figure 2:**
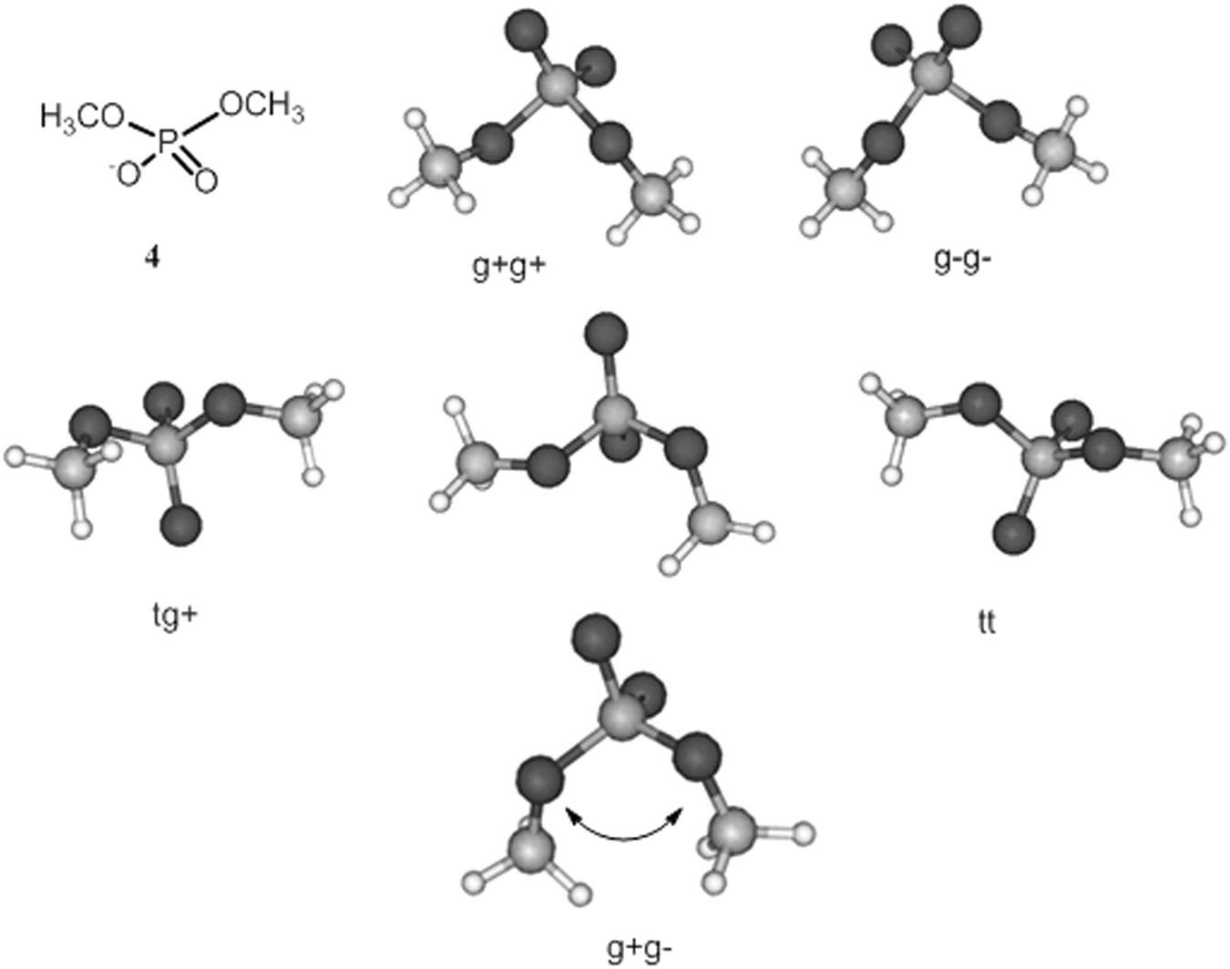
The structure and possible conformations of phosphodiester bridge model **4**.

Prior to performing conformational analysis of disaccharides **1-3**, the general behaviour of the phosphodiester bridge was investigated by using the example of dimethyl phosphate (4) as a simplified model. The conformational state of compound **4** is determined by two C-O-P-O torsional angles, which can have either *gauche* or *trans* orientation (Fig. 2), and gauche conformers can be either *gauche*+ (*g*+) or *gauche*− (*g*−). All possible combinations of these rotamers were constructed as starting conformations. Of course, in the case of such a simple compound, some of them form “*pseudo*-enantiomeric” pairs, but keeping in mind that these fragments would be used further as linkers for asymmetric fragments such as mannoside residues, each of the “*pseudo*-enantiomers” should have been optimized. These starting structures were subjected to geometry optimization by DFT using the B3LYP functional and 6–311 G(2d,2p) basis set. Only one conformation, namely, *g *+* g-*, could be excluded from consideration since it transformed to one of the trans-conformers during the optimization. This result is probably explained by the significant repulsion between two oxygen atoms in such a conformation. The five remaining conformers of the phosphodiester bridge model **4** are shown in Fig. 2.

### Building and modelling the phosphodiester-linked dimannosides 1–3

Using the five conformations of the model phosphodiester **4** described above, several conformations for each of the studied phosphodisaccharides **1**-**3** were built. The methylated oxygen atoms of the linker’s conformers, after deletion of the methyl groups, were used to connect reducing and non-reducing sugar residues. This gave seven possible conformations: Man-(*g *+* g *+)-Man, Man-(*g*−*g*−)-Man, Man-(*g *+* t*)-Man, Man-(*tg *+)-Man, Man-(*g* − *t*)-Man, Man-(*tg*−)-Man and Man-(*tt*)-Man. Additionally, each bond involving atoms forming a glycosidic linkage could have its own conformational preferences. For example, when a non-reducing, or glycosylating, mannose residue was concerned, the torsional angle O5′-C1′-O1′-P was expected to exist in a *gauche* rather than a *trans* orientation due to the exo-anomeric effect^33^. Hence, during the construction of the starting phosphodisaccharide models, the value of this torsion was set to +60°.

As for the residue at the reducing end, or glycosyl phosphorylated residue (i.e., Man-OMe), they are known to have greater rotational freedom. The latter can be described, for example, by the P-Ox-Cx-Hx (x = 3, 4 or 6) torsion angle, which can lie in the region of ±60° or 180°. All three orientations were used, yielding 21 starting conformations for each phosphodisaccharide. These were subjected to molecular dynamics (MD) simulations using an MM3 force field and the Solvent Accessible Surface Area (SASA^34^) approach for water consideration.

**Figure 3:**
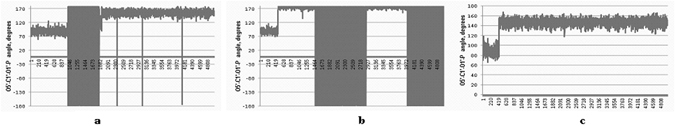
Sample MD trajectories for structures **1**(**a**), **2**(**b**) and **3**(**c**).

In our initial experiments, no counter-ions were included in the simulation. However, analysis of the produced MD trajectories showed that soon after the start of the simulations, all O5′-C1′-O1′-P torsions determining the conformation of the glycosyl phosphate residues changed their orientation to *trans* (sample illustrations of MD trajectories are given in Fig. 3). Since that contradicted the expectations arising from the well-known *exo*-anomeric effect^33, 35^, a literature search was performed. Tvaroska *et al*.^20^ observed a similar effect during *ab initio* modelling of tetrahydropyranyl methyl phosphate. In particular, when the calculations were performed in the absence of a cation, the conformation with the lower energy was also that having O5′-C1′-O1′-P torsion in the *trans* orientation (reverse *exo*-anomeric effect^36^). When a sodium atom was included, the expected situation (result of the *exo*-anomeric effect) was restored with the O5′-C1′-O1′-P torsion having a *gauche* orientation. Tvaroska^20^ speculated that this was probably due to the ability of Na^+^ not only to shield the negative charge on the phosphate bridge but also to coordinate with the intra-ring oxygen O5′.

However, (1 → 3)- and (1 → 4)-linked compounds **1** and **2** were primarily investigated in their triethylammonium forms. It was unclear using general considerations to deduce whether the NHEt_3_^+^ cation could have the same influence on the conformation of the O5′-C1′-O1′-P torsion as the Na^+^ cation. The situation had additional complications such as hydroxyl groups from the other mannoside residue, which could also interact in some fashion with the NHEt_3_^+^ or Na^+^ moieties. Hence, another series of DFT calculations was performed for (1 → 3)- (**1**) and (1 → 4)-linked (**2**) compounds with the NHEt_3_^+^ cation and for the (1 → 6)-linked disaccharide **3** with Na^+^ as the counter-ion. This time, two different starting conformations were used for O5′-C1′-O1′-P torsion of the glycosylating mannose, either *gauche* or *trans*. The conformations of the rotatable bonds in the phosphate bridge were the same as described above.

In all of the performed DFT calculations, it was found that the NHEt_3_^+^ cation indeed produced the same conformational effect as Na^+^, i.e., in all cases, structures with the *gauche* orientation of the O5′-C1′-O1′-P torsion in accordance with the *exo*-anomeric effect were found to have lower energies. Thus, the quantum mechanical calculation results led to a conclusion that shielding the negative charge on the phosphate oxygens played a greater role than any plausible additional interactions with O5′ or other fragments of the studied molecules.

With these results obtained, MD simulations were performed again on all the conformers with inclusion of the counter-ions. Additionally, restraints on O5′-C1′-O1′-P torsion stemming from DFT calculations were applied to hold it in the *gauche* conformation determined by the *exo-*anomeric effect. The resulting ensembles of the conformers were analysed to measure their average inter-atomic distances and torsional angles of interest to compute NOE and coupling constants.

### Nuclear Overhauser effect (NOE) analysis

To estimate the theoretical NOE values, corresponding inter-unit proton-proton distances were used. They were measured in each of the snapshot conformations and then averaged. Although averaged values of r^−6^ are required for the quantitative calculations of NOE, some assumptions about the presence or absence of the effect for certain proton pairs can be based on the fact that the NOE is measurable only in case when the inter-proton distance is less than 4.5 Å.

**Figure 4:**
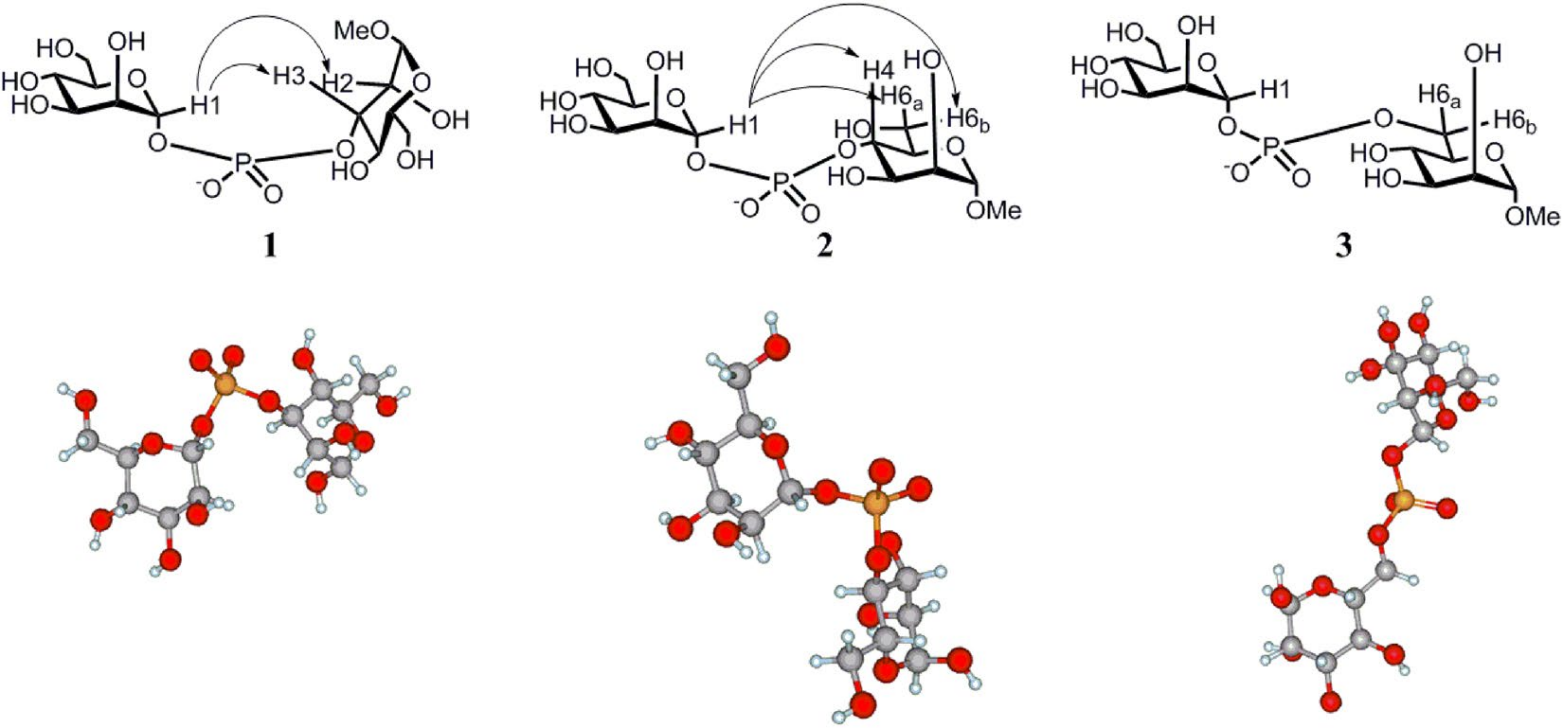
Illustration of the observed NOEs (shown by arrows) in compounds **1**-**3** upon pre-irradiation of the H1′ proton in the glycosylating residue at 303 K.

**Figure 5:**
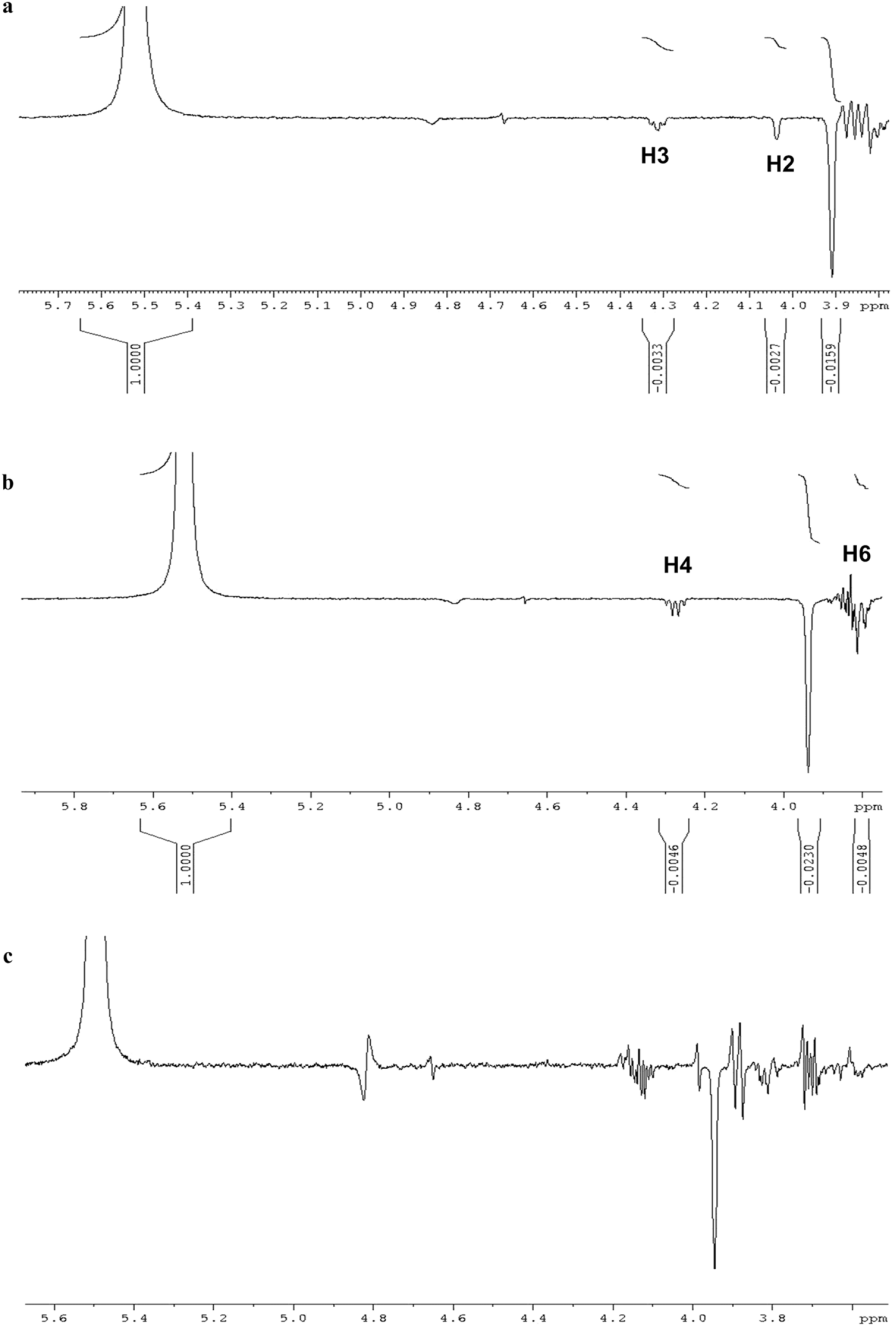
Fragments of 1D NOESY spectra for compounds **1**(A), **2**(B) and **3**(**C**).

Experimental data were obtained by means of 1D NOESY. During the analysis of the MD snapshots, it was found that while the glycosylating section was initially restrained from rotation around the C1′-O1′ bond, the glycosyl phosphorylated section (i.e., Man-OMe) demonstrated a high degree of conformational flexibility. However, in the case of the (1 → 3)-linked structure **1** and the (1 → 4)-linked structure **2**, this flexibility led to the possibility of the studied molecules adopting conformations in which the respective inter-ring H1′-H3 and H1′-H4 distances became lower than 4 Å and their averaged values were in the range of 3–3.5 Å, signifying the possibility of NOE (Table 3, Fig. 4). In case of (1 → 4)-linked compound **2**, the distances H1′-H6_a_ and H1′-H6_b_ were also small enough to suggest NOE. In addition, in this case some NOE was indeed observed on H6 protons (Fig. 5b). Similarly, NOE was observed on the H2 proton of (1 → 3)-linked compound **1** (Fig. 5a and Table 3). This explained the appearance of inter-ring NOEs in the case of compounds **1** and **2** (Figs 4 and 5).

**Table 3:** Calculated averaged inter-proton distances in compounds **1–3**.

Compound	Atom pair	Distance, Å
**1**	H1′-H3	3.5
	H1′-H2	3.3
**2**	H1′-H4	3.1
	H1′-H6_a_	3.6
	H1′-H6_b_	3.6
**3**	H1′-H6_a_	4.8
	H1′-H6_b_	4.7

Quantitative analysis of the observed NOE values was attempted. The H1′-H2′ distance that was measured to be approximately 2.6 Å was used as a reference. Considering that relative NOEs are proportional to the reciprocal sixth power of inter-proton distances, this suggested that in compounds **1** and **2**, the H1′-H2′ NOE should have values 3 to 6 times larger than those of the inter-unit effects. From Fig. 5a,b it can be seen that these proportions are all 5x. This means that no exact quantitative correlation could be established in this case.

Unlike compounds **1** and **2**, for (1 → 6)-linked molecule **3**, the presence of an extra C6-C5 bond near the phosphodiester bridge leads to a spatial separation of the two pyranoside residues that could not be overcome by the enhanced conformational flexibility of the P-O6-C6-C5 fragment. The averaged distances in compound **3** between H1′ and the protons H6 of the pyranoside section in the Man-OMe unit exceeded 4 Å (Table 3). In fact, no reliable NOE effects were observed experimentally in this instance (Fig. 5c).

Additionally, the temperature dependence of the NOE effects was studied for compounds **1** and **2** (as their ammonium salts) to gain further insight into their conformational equilibrium. 1D NOESY experiments were performed for these substances at temperatures of 263, 283, 303 and 323 K in methanol-*d4* solutions. At the lowest temperature (263 K), no NOE were observed, most likely because of the total decrease in molecular motion. 1D ROESY spectroscopy was attempted in this case, but since the quantitative integration of ROE signals may give significant errors, these values are not shown; the corresponding spectra are presented in Supplementary Information.

At higher temperatures, different tendencies were found for the investigated structures (Table 4). For (1 → 4)-linked compound **2**, no detectable changes were observed in H1′-H4 NOE with increasing temperature, while the strength of the H1′-H6_a,b_ signal increased. Some slight changes occurred in (1 → 3)-linked molecule **1**: a relative decrease in H1′-H2 NOE along with a simultaneous slight increase in H1′-H3. This confirms, in our opinion, that the conformational distribution in the studied structures has a rather complex nature due to the relative flexibility of the phosphate linkage.

**Table 4:** NOE values (relative to H1′-H2′ NOE value) at different temperatures for compounds **1** and **2**.

Compound	Atom pair	283 K	303 K	323 K
**1**	H1′-H3	0.13	0.14	0.14
	H1′-H2	0.11	0.09	0.09
**2**	H1′-H4	0.18	0.18	0.18
	H1′-H6_a,b_	0.24	0.24	0.33

### Analysis of three-bond ^13^C-^31^P and H-^31^P coupling constants (^3^*J*_C,P_ and ^3^*J*_H,P_)

^3^ *J*_C,P_ as well as other three-bond coupling constants can be calculated empirically using a specially parametrized Karplus-type equation. Equation (1), given in work^37^, was used in the present study:

1 $${}^{3}J_{{\rm{CCOP}}}=6.9{\cos }^{2}{\rm{\phi }}-3.4\,\cos \,{\rm{\phi }}+0.7$$

where φ defines the corresponding torsional angle C-C-O-P. These torsions and subsequent coupling constants were calculated for each of the snapshot conformations to evaluate theoretical ^3^ *J*_C,P_ values. The averaged values of these constants were than compared to the experimental ones that are readily measured from 1D ^13^C NMR spectra (Table 5).

**Table 5:** Experimental and calculated (in parenthesis) averaged three-bond coupling constants 3JC,P for compounds **1–3**.

Compound	Interacting atoms	3JC,P values, Hz
**1**	P-C2	2.7 (1.5)
	P-C4 P-C2′	4.4 (3.9) 9.2 (8.5)
**2**	P-C3	<2 (1.5)
	P-C5 P-C2′	7.1 (6.1) 7.4 (6.7)
**3**	P-C5 P-C2′	7.8 (6.9) 7.9 (7.2)

A generally good coincidence can be observed between the theoretical and the calculated data. However, the deviation sometimes reaches 1 Hz. This can be explained by the already mentioned fact that the studied molecules demonstrate high conformational flexibility, especially at the glycosyl phosphorylated end, which leads to a greater error in the computed constants.

Since no reliable NOE were observed for (1 → 6)-linked phosphodimannoside **3**, an additional investigation that consisted of the measurement of ^1^H-^31^P three-bond couplings was performed. For their theoretical calculation, another Karplus-type equation (2) was employed, taken from work^38^:

2 $${}^{{\rm{3}}}{\rm{J}}_{{\rm{HCOP}}}=15.3\,{\cos }^{2}{\rm{\phi }}-6.1\,\cos \,{\rm{\phi }}+1.6$$

Table 6 shows the experimentally measured ^3^J_H,P_ constants in comparison with those calculated as averages of the ensemble of conformers as described above for the ^3^J_C,P_. Unlike the P-C constants that were generally underestimated in calculations, P-H couplings tended to be largely (more than 1 Hz) overestimated. We believe this can result from the used Karplus-type equation (2) having been originally derived for nucleotides such that it may give larger errors in the case of phosphodimannosides, although the general trend is reproduced.

**Table 6:** Experimental and calculated (in parenthesis) averaged three-bond coupling constants 3JH,P (Hz) in the 1 H NMR spectra of compounds **1–3**.

Compound	Interacting atoms	3JH,P values, Hz
1	P-H1′	7.6 (8.8)
	P-H3	8.0 (8.9)
2	P-H1′	7.3 (8.1)
	P-H4	9.0 (9.5)
3	P-H1′	7.7 (8.8)
	P-H6a	5.9 (6.8)
	P-H6b	6.8 (8.0)

## Conclusions

The conformations of numerous di- and trisaccharides (i.e., conformations around various glycoside bonds) have been thoroughly examined over the last 30 years. In contrast, the conformational properties of phosphodiester-linked disaccharides have not been studied to date. To fill this gap, we conducted the first conformational study of oligosaccharides bearing phosphodiester bridges.

The conformational behaviour of a series of phosphodiester-bridged dimannosides was studied by means of NMR spectroscopy, DFT calculations and MD simulations using a continuum solvation model and the MM3 force field. When the presence of a counter-ion was neglected, the reverse *exo*-anomeric effect occurred at the glycosylating part of the molecules, most likely due to the repulsion between the negative charges on the phosphate group and the saccharide oxygen atoms. When either sodium or triethylammonium cations were added, the standard conformation determined by the *exo-*anomeric effect was restored. In general, the studied molecules demonstrated a high degree of conformational flexibility, which in the case of (1 → 3)- and (1 → 4)-linked structures led to the spatial proximity of H1′ and Hx atoms resembling (in some extent) that one in common disaccharides linked by a glycosidic bond.

## Methods

The NMR spectra were recorded on a Bruker Avance 600 spectrometer for solutions in CD_3_OD at 30 °C or other temperatures when specified. TSP (δ_H_ 0.0 and δ_C_ −1.6 ppm) was used as the internal standard for ^1^H and ^13^C spectra, and 85% H_3_PO_4_ (δ_P_ 0.0) was used as an external standard for ^31^P spectra. The 2D NMR spectra were recorded and treated according to the standard Bruker software. A mixing time of 60 ms was used in TOCSY experiments. 1D ROESY spectra were registered with cw spinlock for mixing using refocusing with a shaped pulse. The spinlock time was 0.2 s. 1D NOESY spectra were registered using selective refocusing with a shaped pulse. The spinlock time was 0.7 s.

DFT calculations were performed using DALTON2015^39^ software. Optimizations were performed *in vacuo* until the gradient reached values less than 10^−4^. Molecular mechanics calculations were performed using the TINKER 5.0^40^ software package with the MM3 force field. The Solvent Accessible Surface Area (SASA) continuum solvation model was used in all cases. Each MD trajectory length was 50 ns with the snapshot conformations being written each 2 ps, thus producing 25000 structures for each simulation. Geometrical constraints were applied using the standard algorithm provided by the software using force constants of 5.

1D ROESY and 1D NOESY spectra recorded at different temperatures are available in the Supplementary file. Other experimental data used in this study are available from the authors upon request.

## Electronic supplementary material

Supplementary Information
