# Supplementary material for: Theoretical and NMR-based Conformational Analysis of Phosphodiester-linked Disaccharides
Source: Sci Rep. 2017 Aug 21;7:8934. doi: 10.1038/s41598-017-09055-x (PMC5566550; doi:10.1038/s41598-017-09055-x)
Supplement: Supplementary file 1 — Supplementary Information [file 41598_2017_9055_MOESM1_ESM.pdf]

Supplementary Information

Theoretical and NMR-based Conformational Analysis of Phosphodiester-linked Disaccharides

Alexey G. Gerbst, Andrei V. Nikolaev, Dmitry V. Yashunsky, Alexander S. Shashkov, Andrey S. Dmitrenok, Nikolay E. Nifantiev.

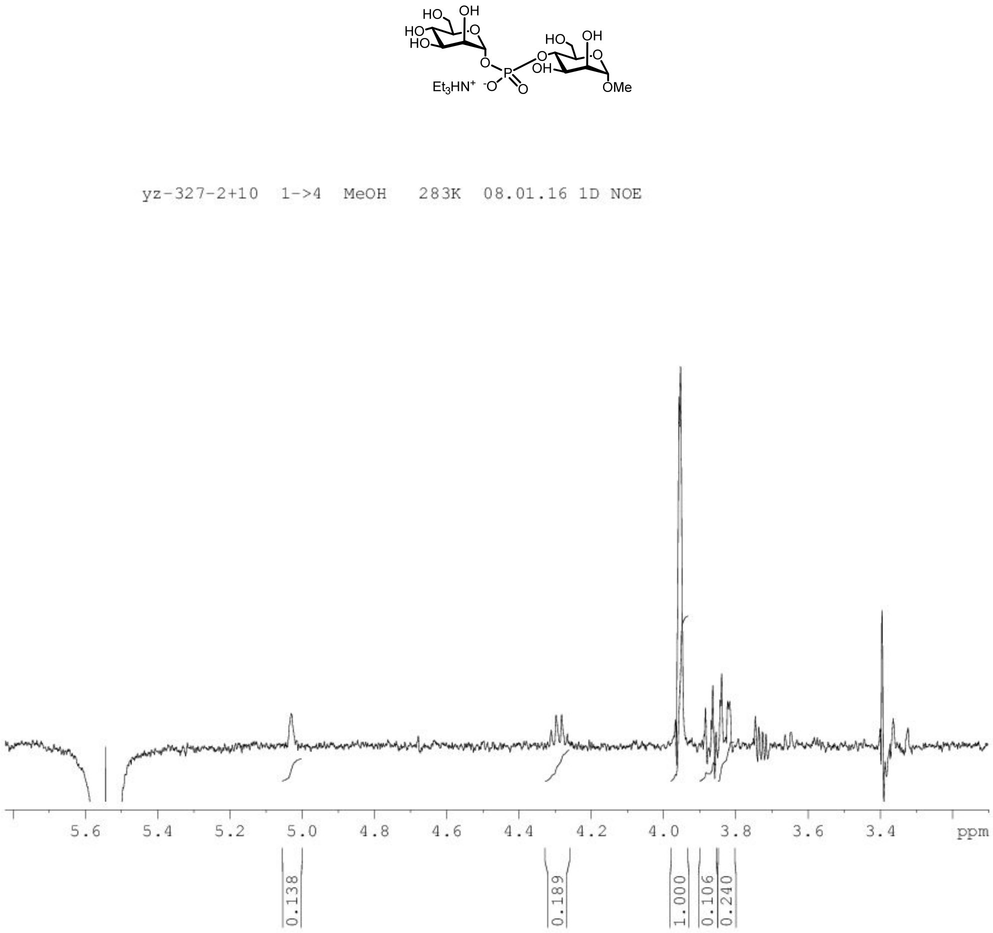

Figure S1. 1D NOESY spectrum of compound **2** at 283 K.

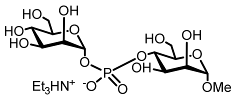

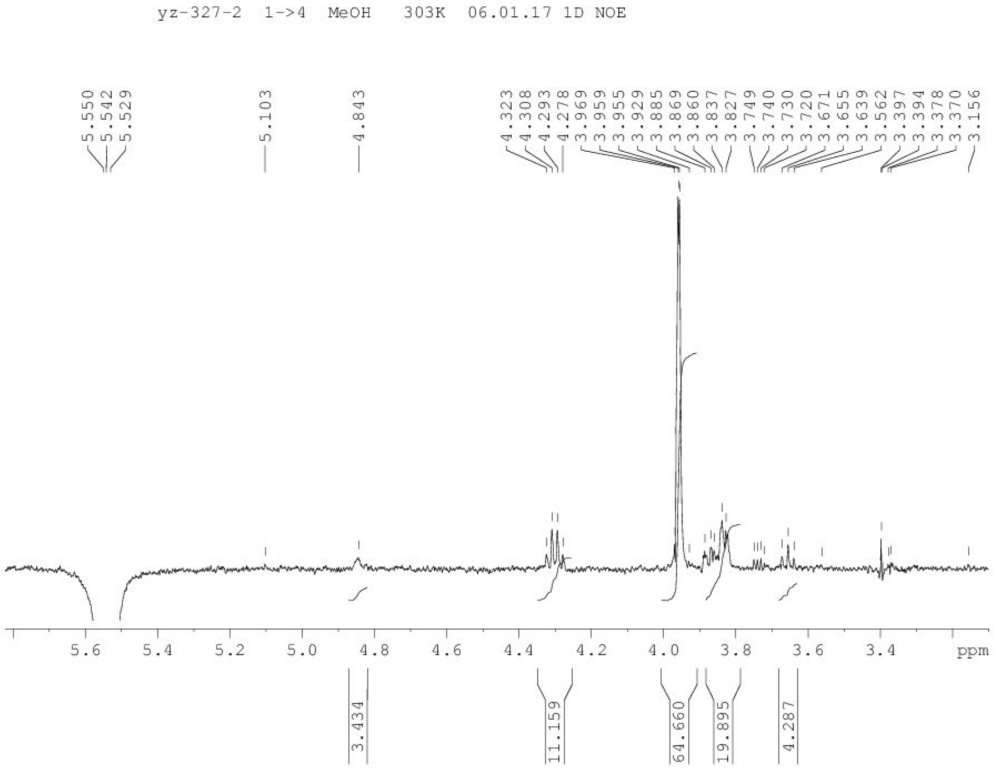

Figure S2. 1D NOESY spectrum of compound **2** at 303 K.

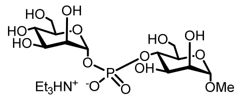

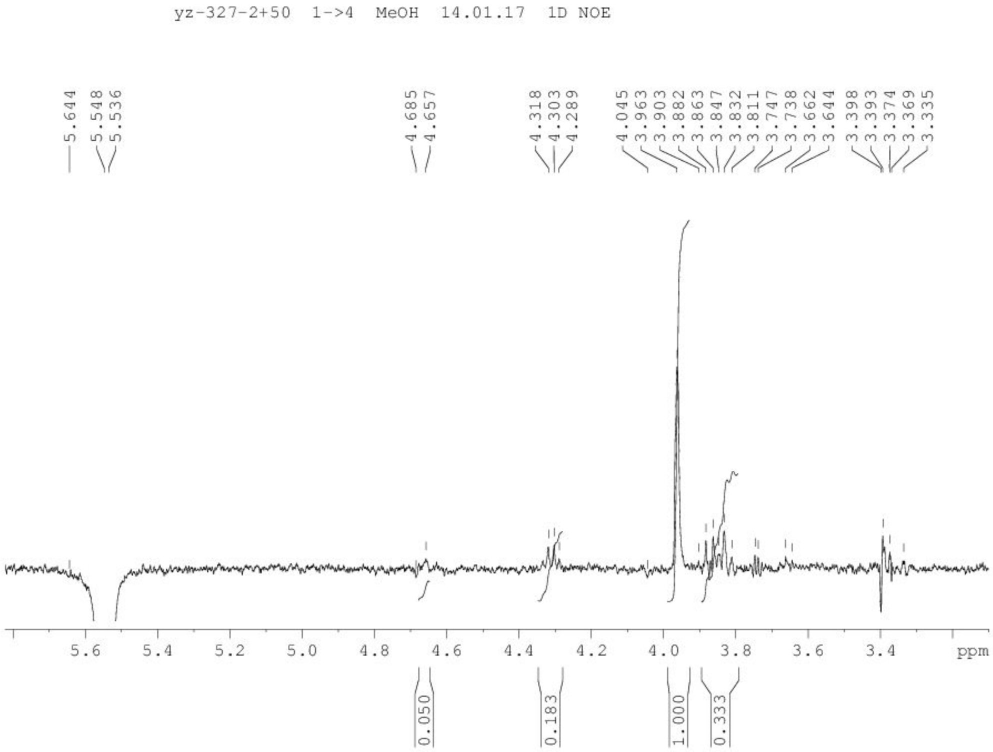

Figure S3. 1D NOESY spectrum of compound **2** at 323 K.

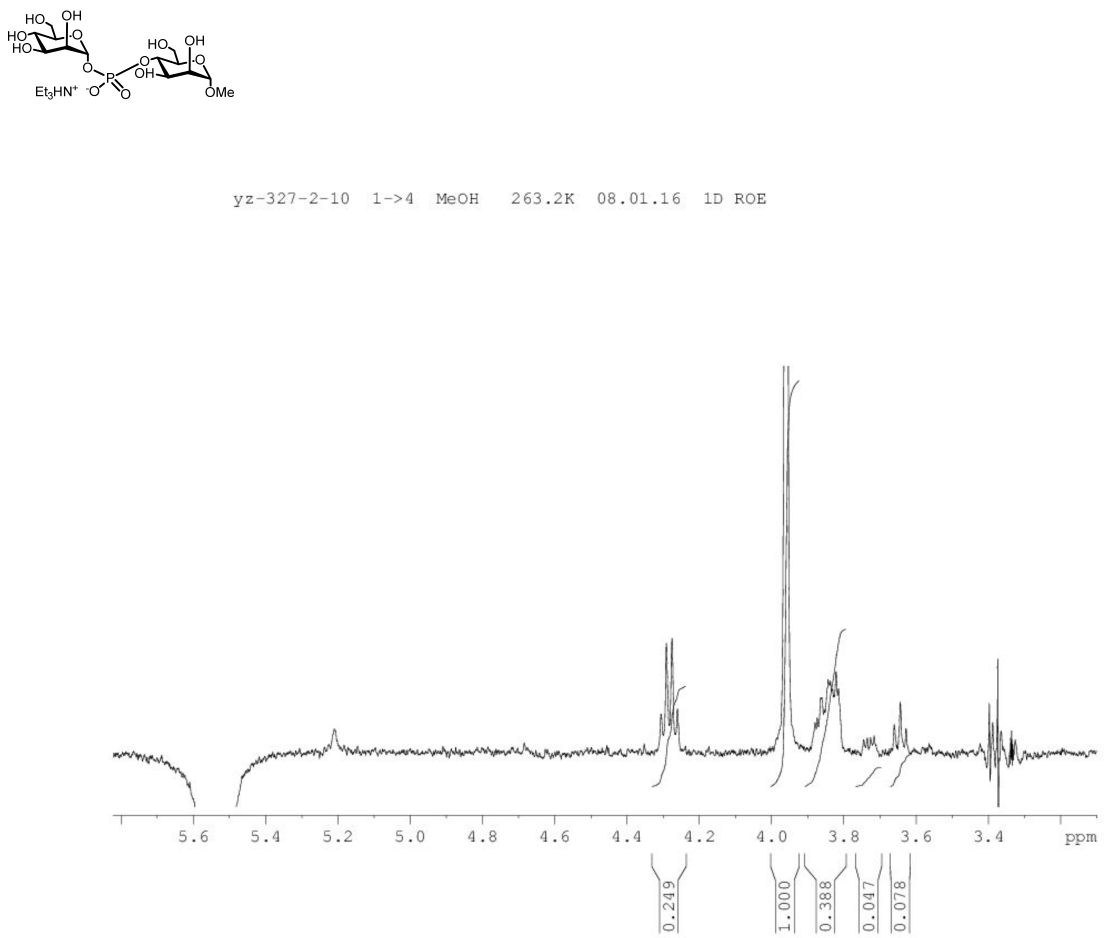

Figure S4. 1D ROESY spectrum of compound **2** at 263 K.

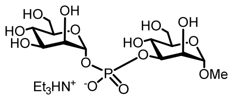

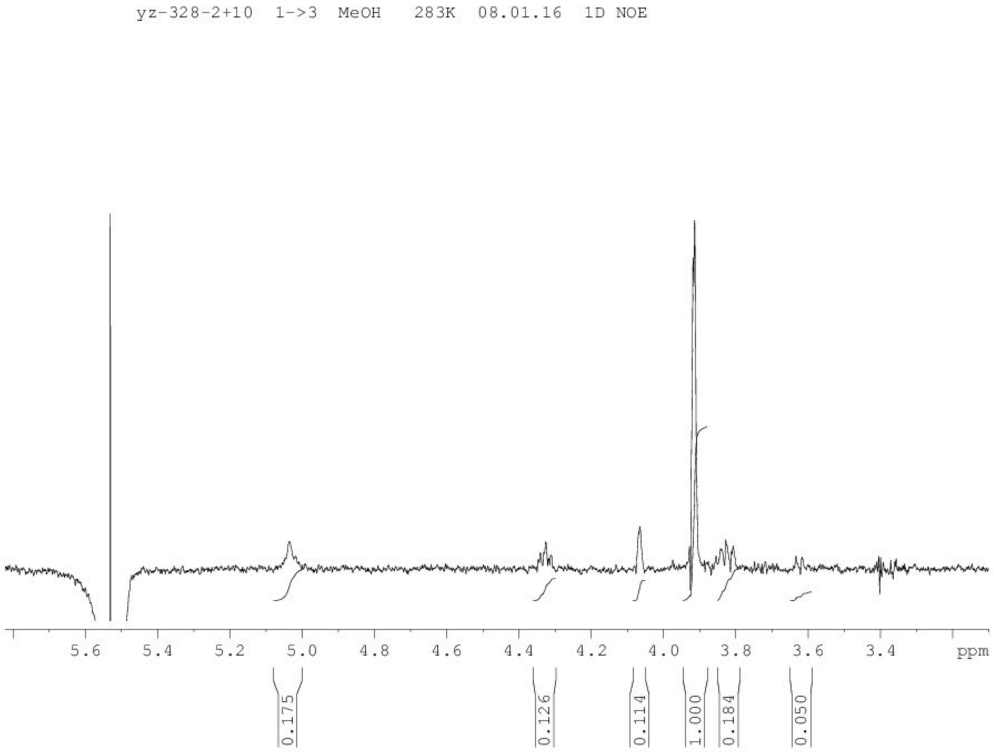

Figure S5. 1D NOESY spectrum of compound **1** at 283 K.

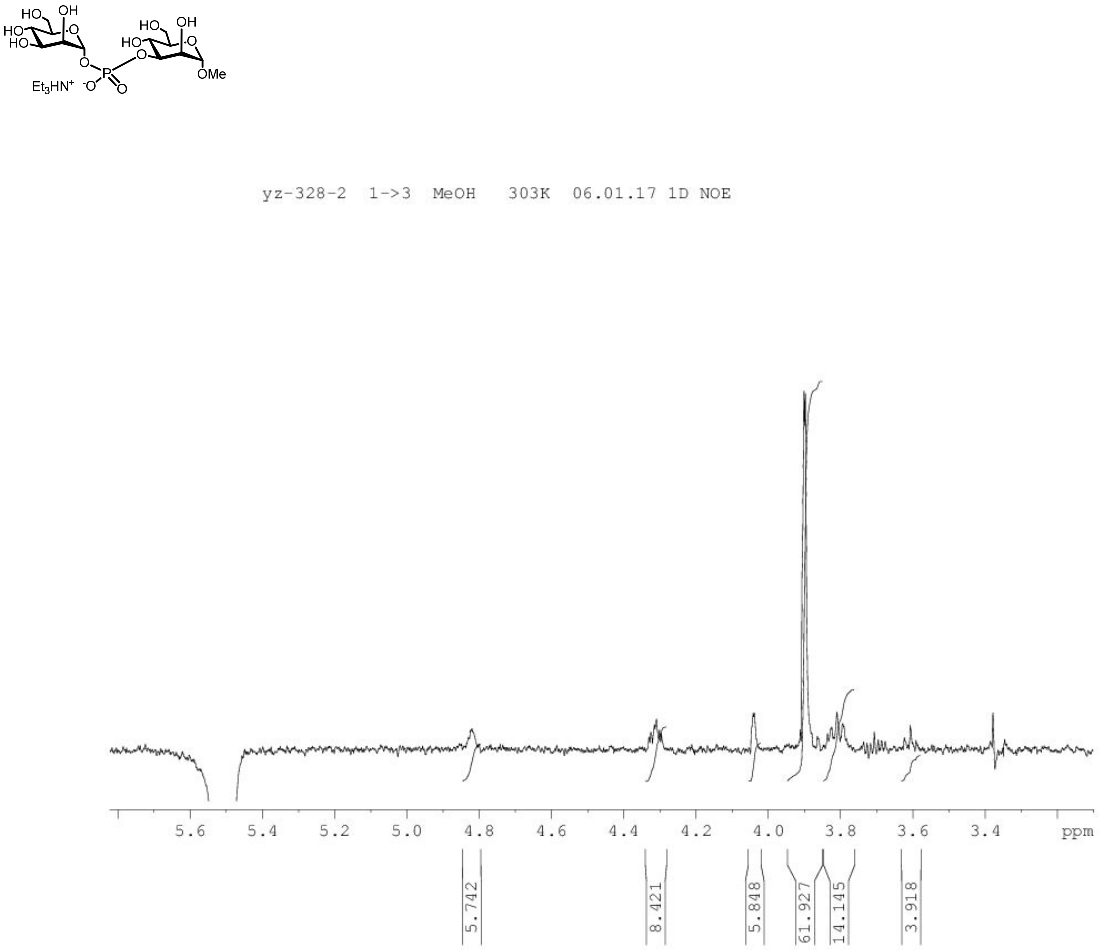

Figure S6. 1D NOESY spectrum of compound **1** at 303 K.

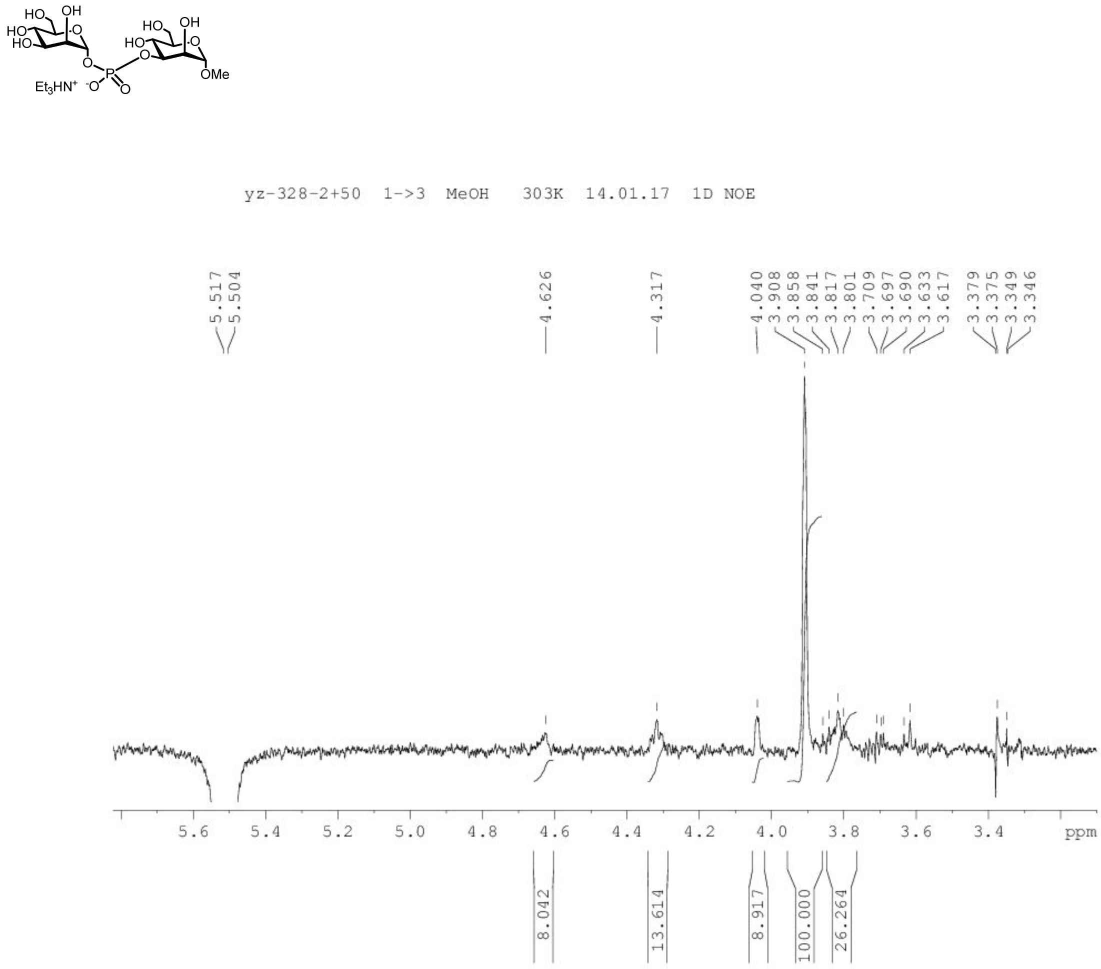

Figure S7. 1D NOESY spectrum of compound **1** at 323 K.

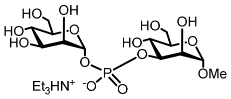

1D ROESY at 263 K.

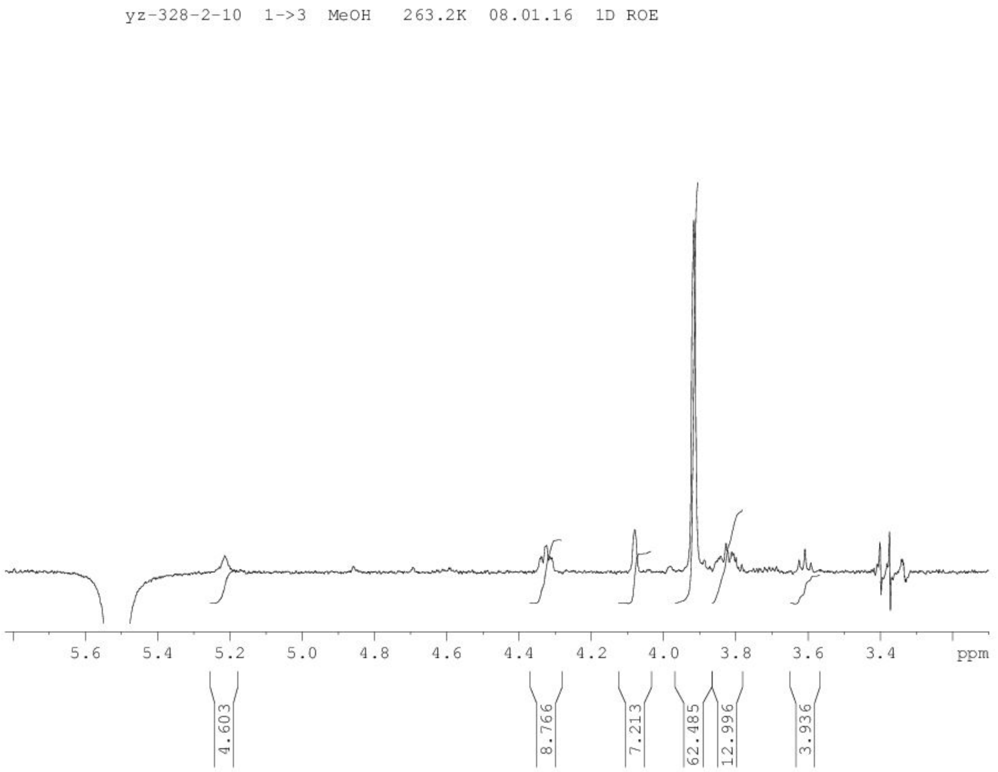

Figure S8. 1D ROESY spectrum of compound **1** at 263 K.

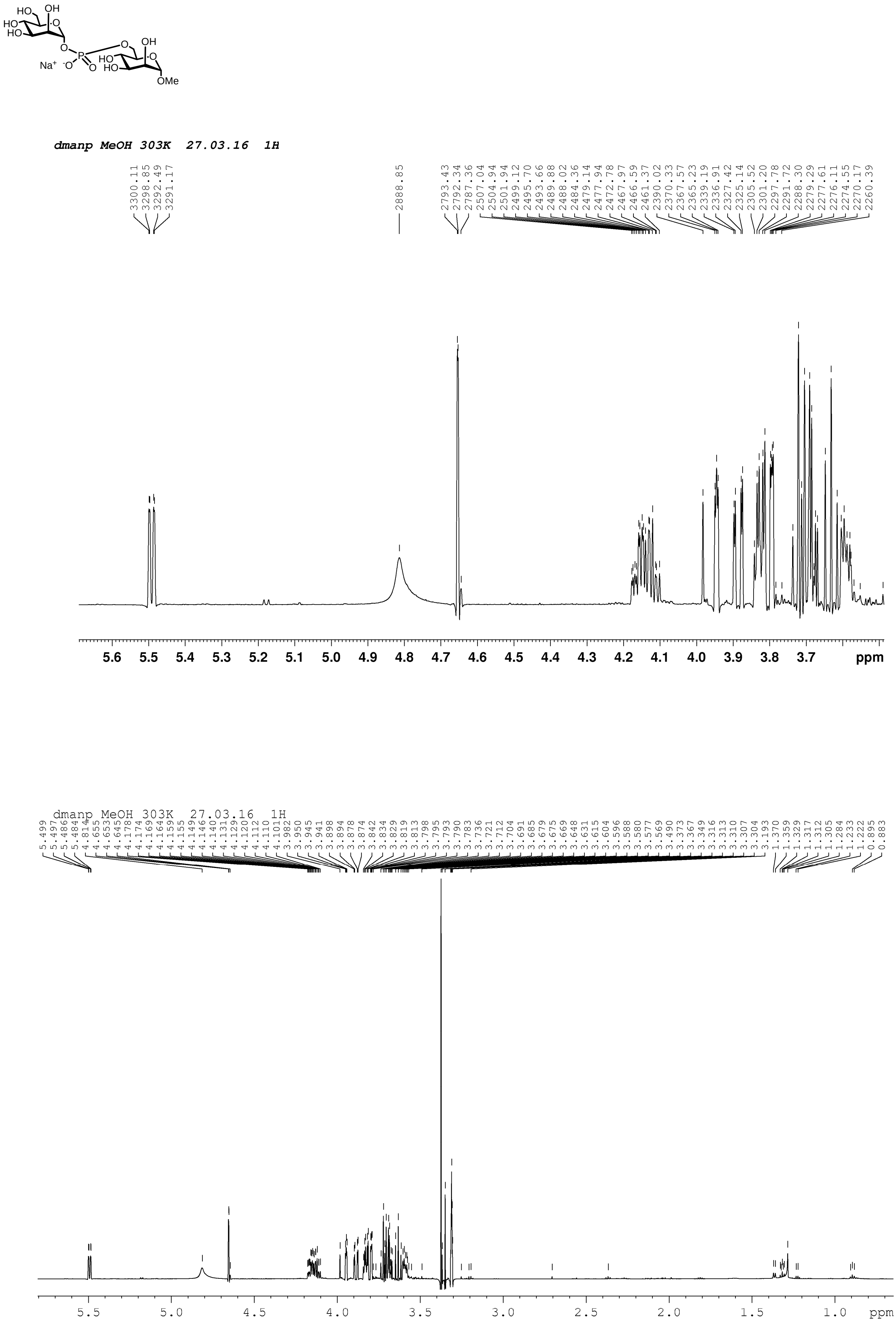

Figure S9. ^{1}H NMR spectra for compound **3**.

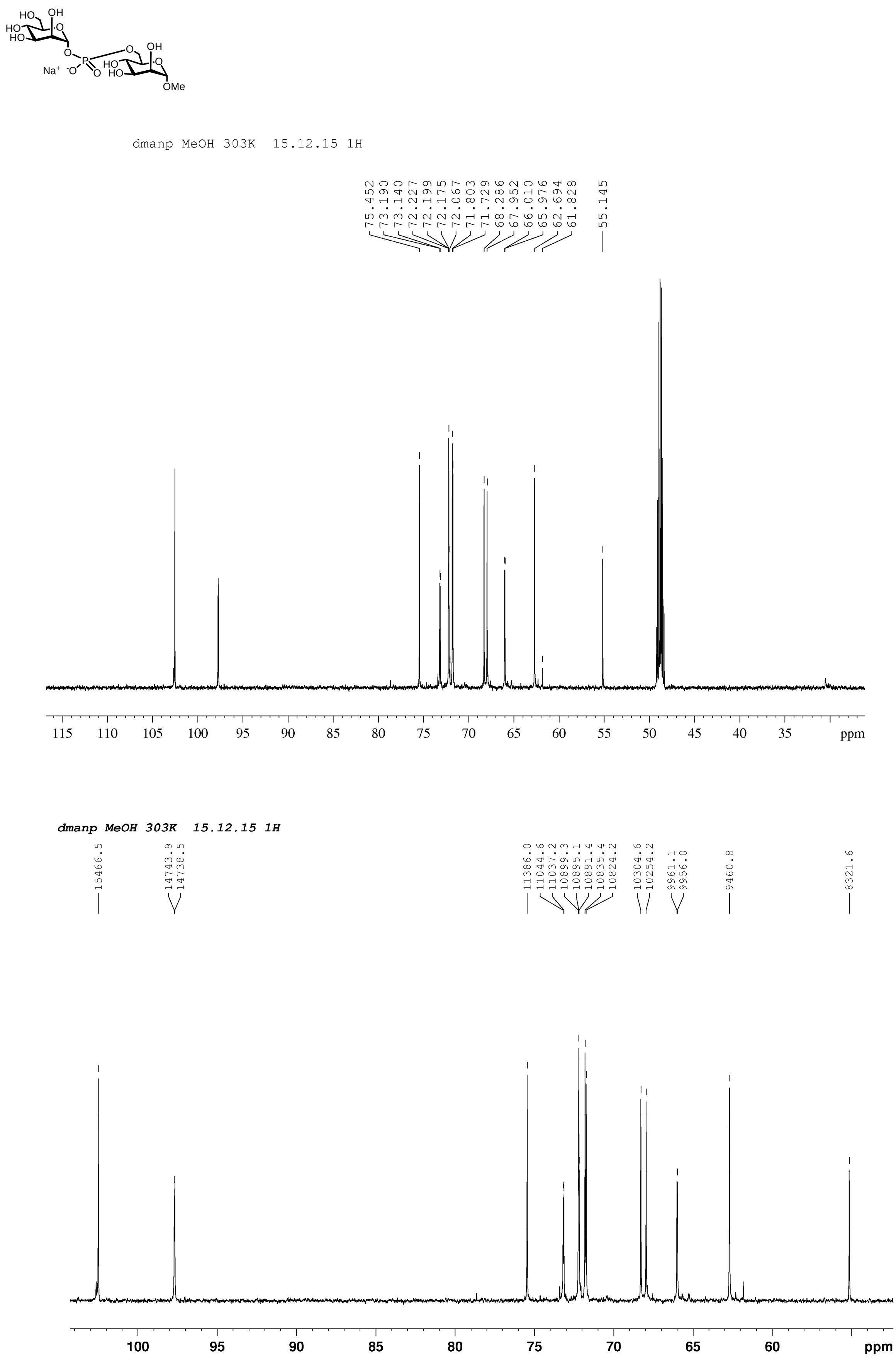

Figure S10. ^{13}C NMR spectra for compound **3**.

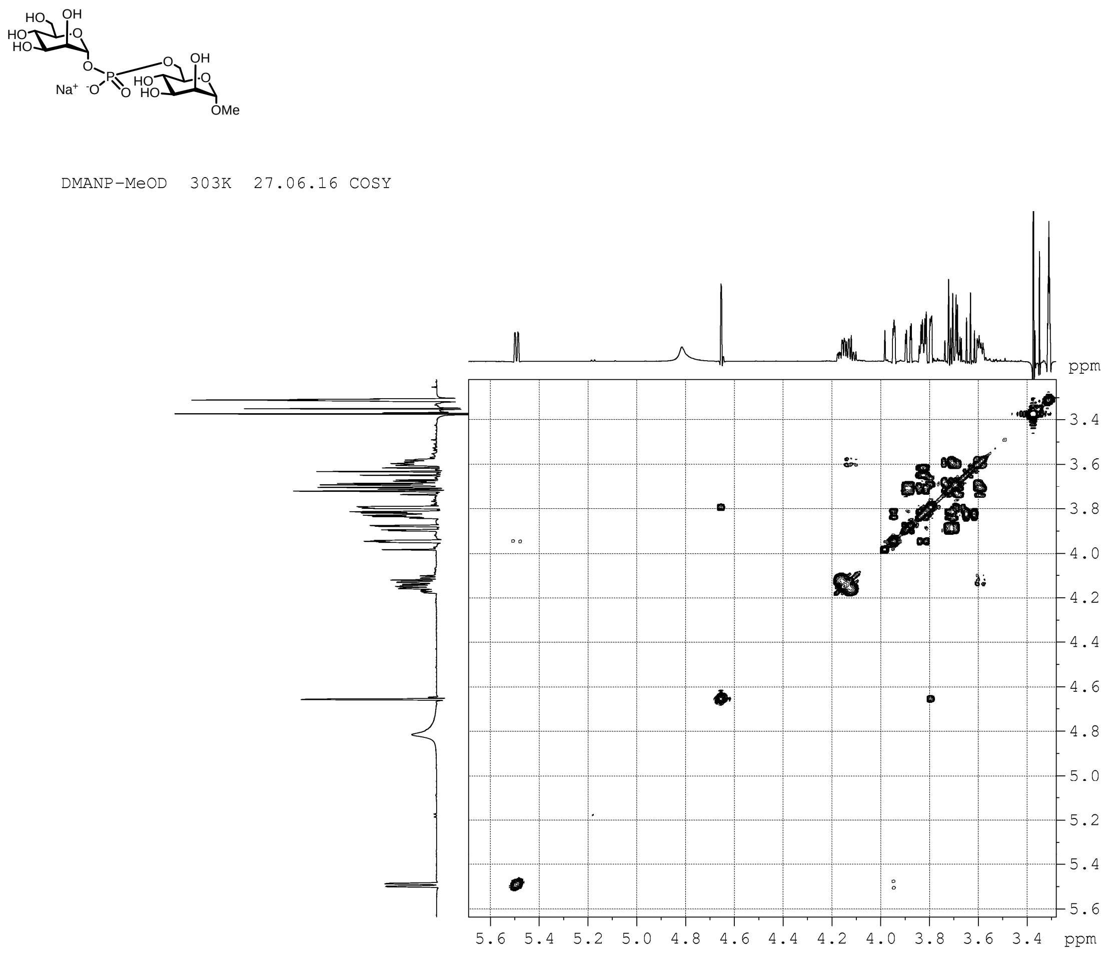

Figure S11. ^{1}H-^{1}H COSY spectrum for compound **3**.

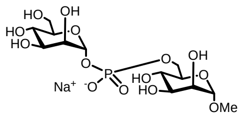

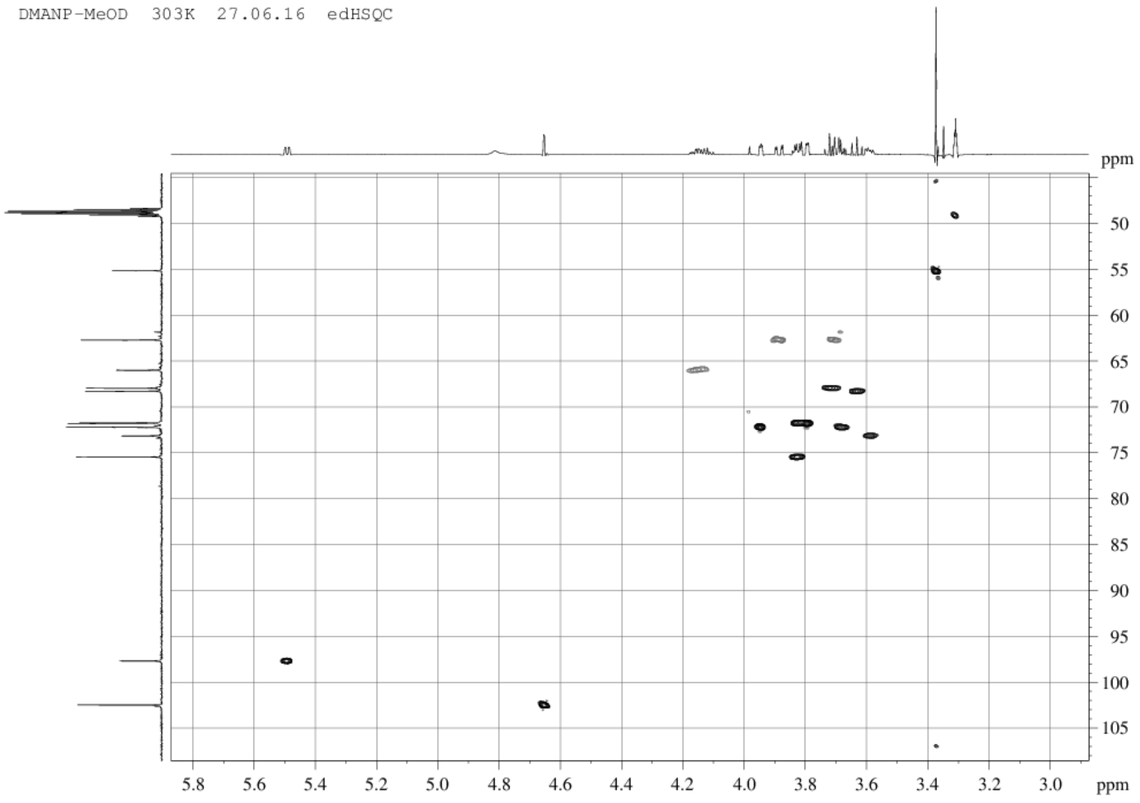

Figure S12. ^{1}H-^{13}C edHSQC spectrum for compound **3**.

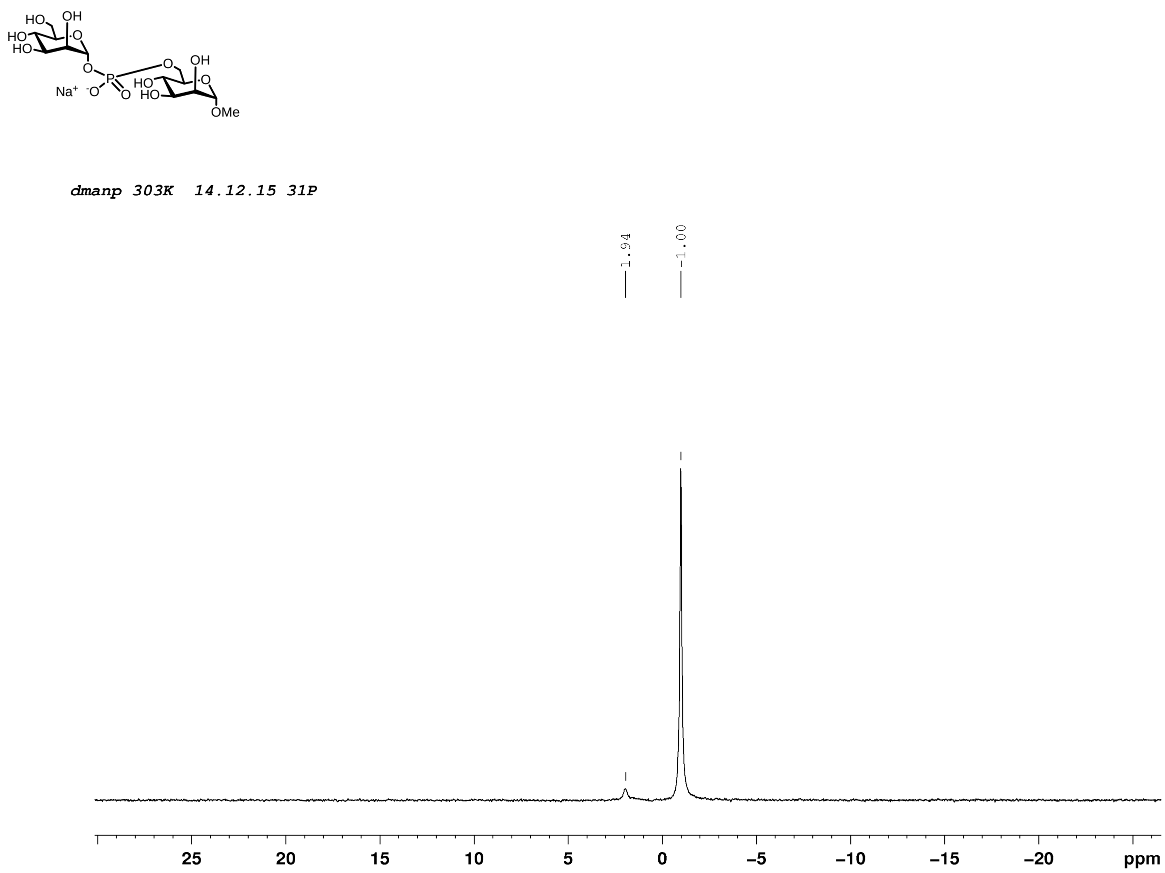

Figure S13. ^{31}P NMR spectrum for compound **3**.

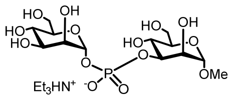

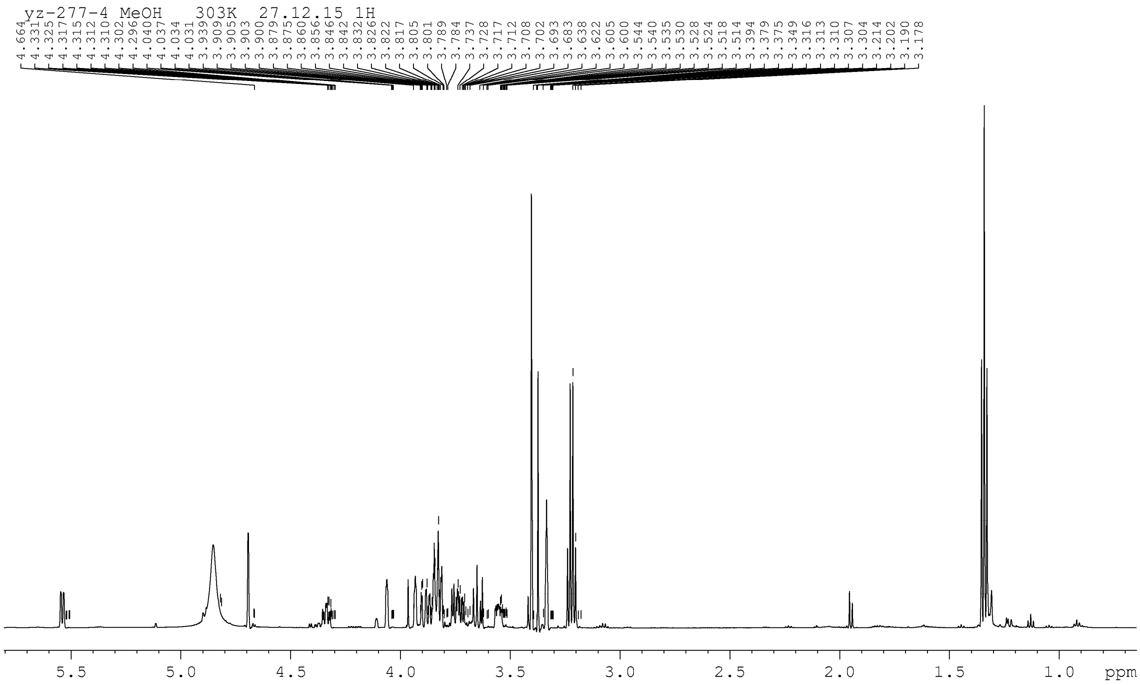

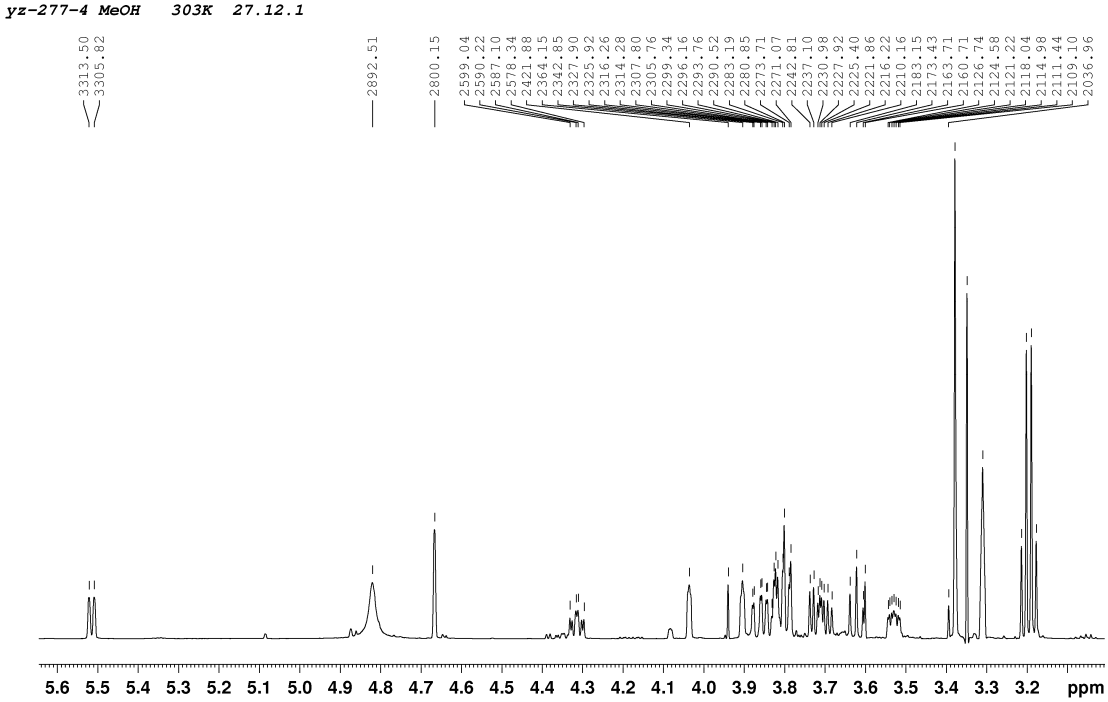

Figure S14. ^{1}H NMR spectra for compound **1**.

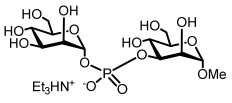

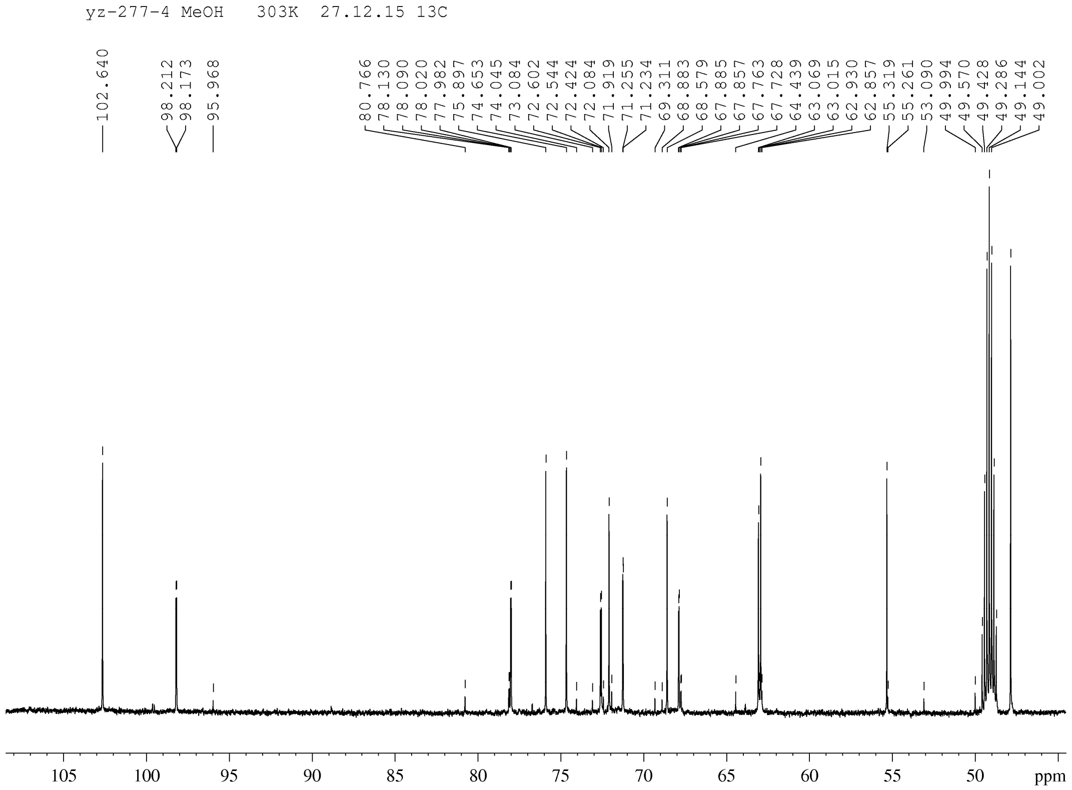

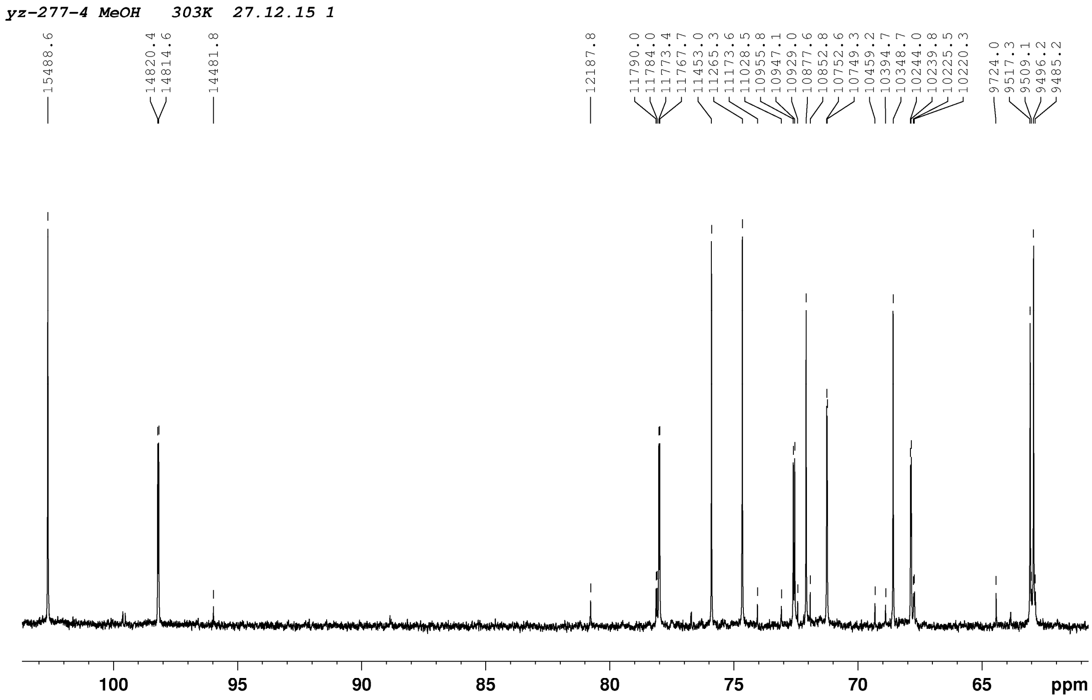

Figure S15. ^{13}C NMR spectra for compound **1**.

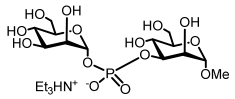

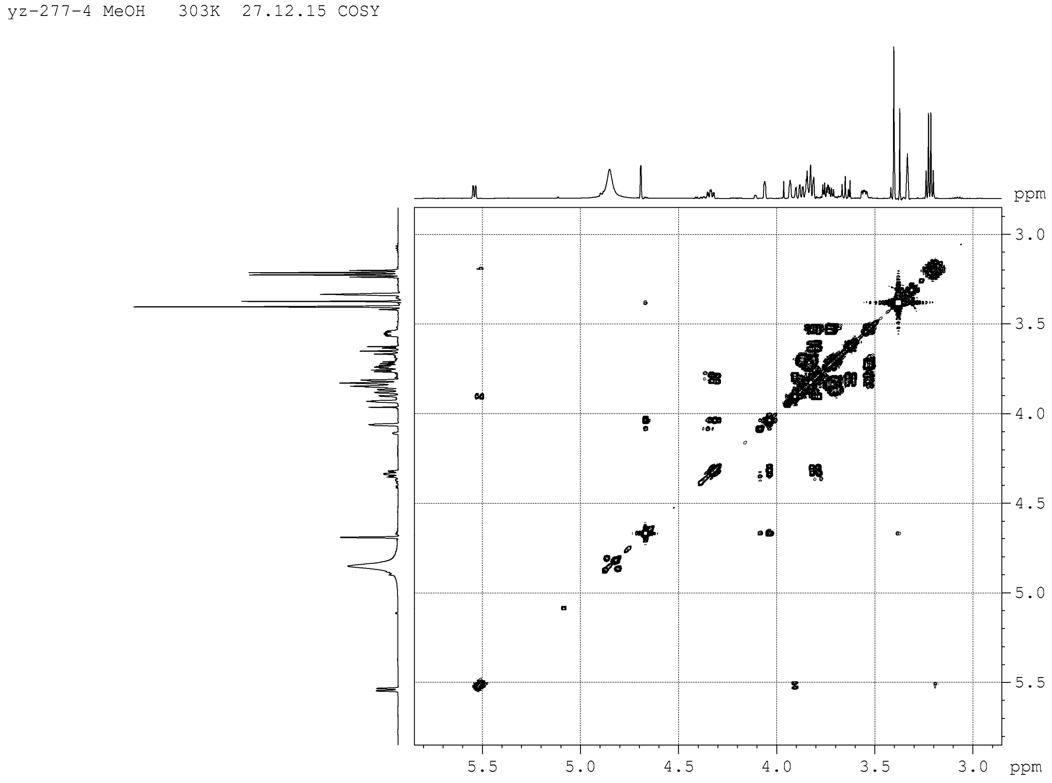

Figure S16. ^{1}H-^{1}H COSY spectrum for compound **1**.

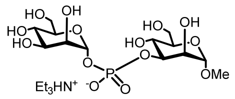

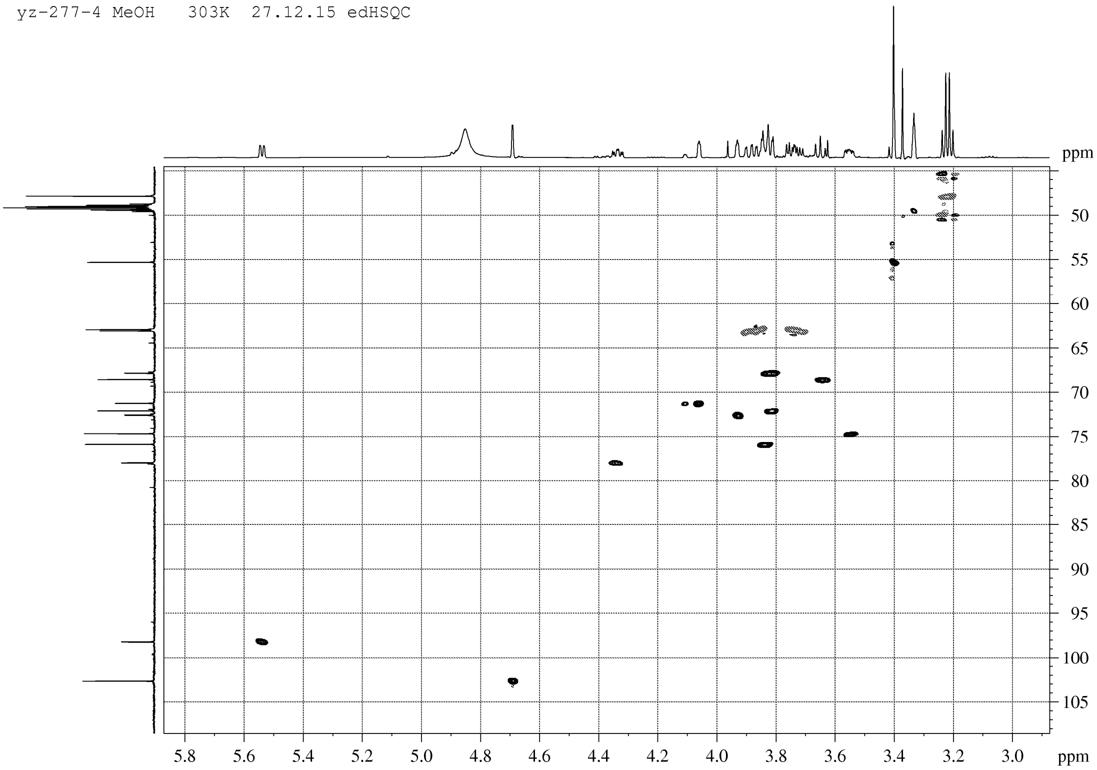

Figure S17. ^{1}H-^{13}C edHSQC spectrum for compound **1**.

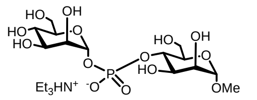

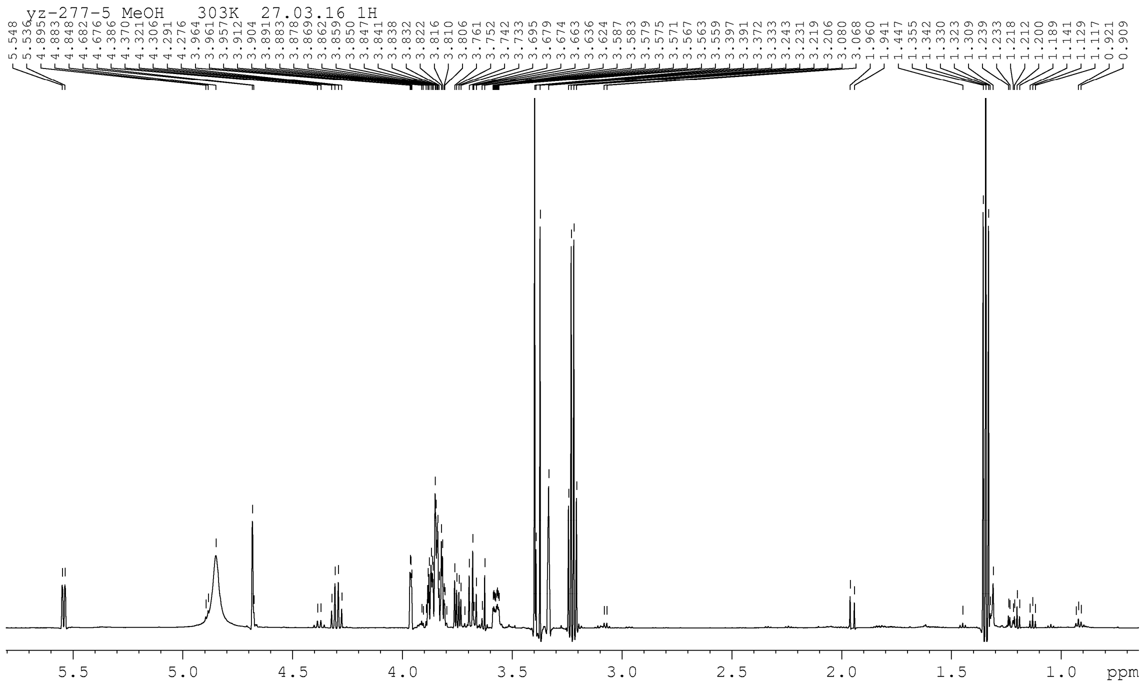

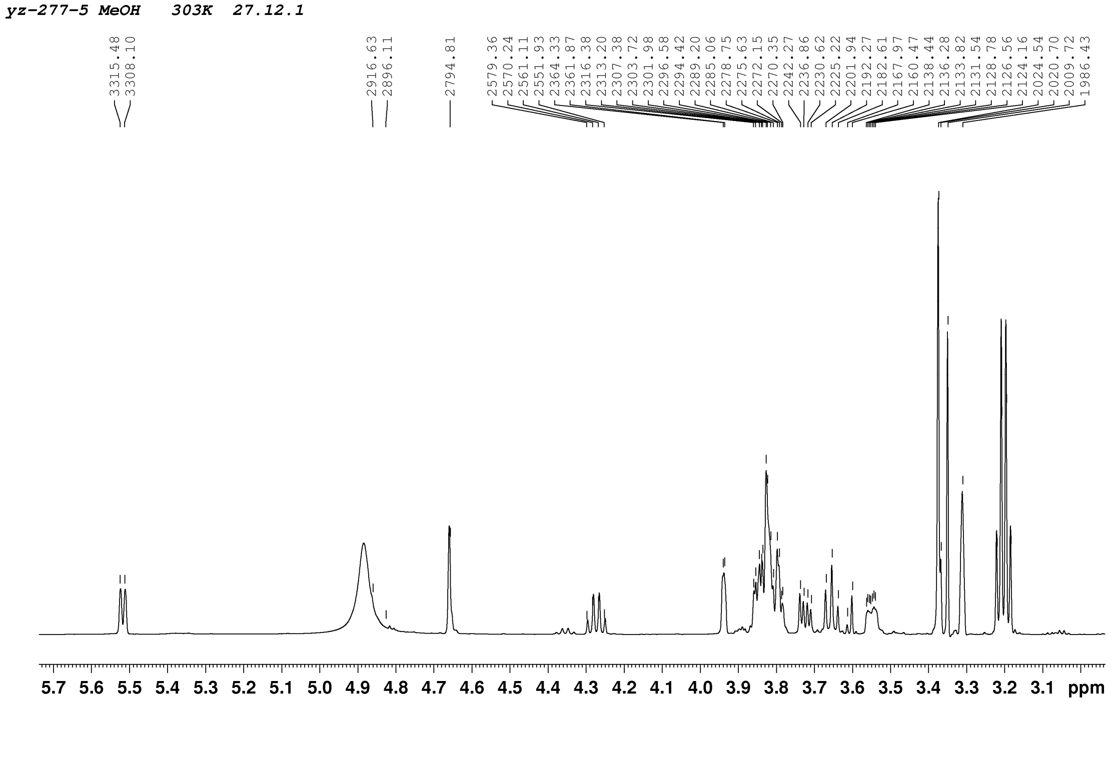

Figure S18. ^{1}H NMR spectra for compound **2**.

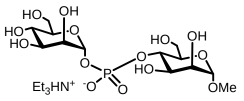

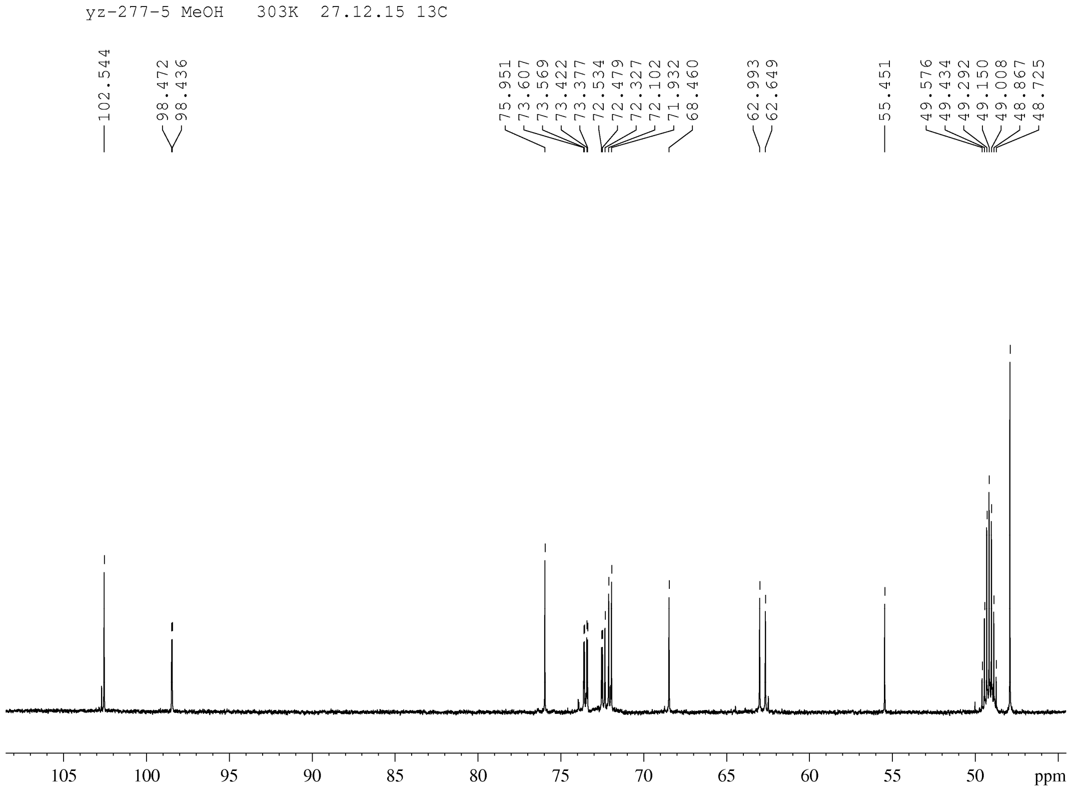

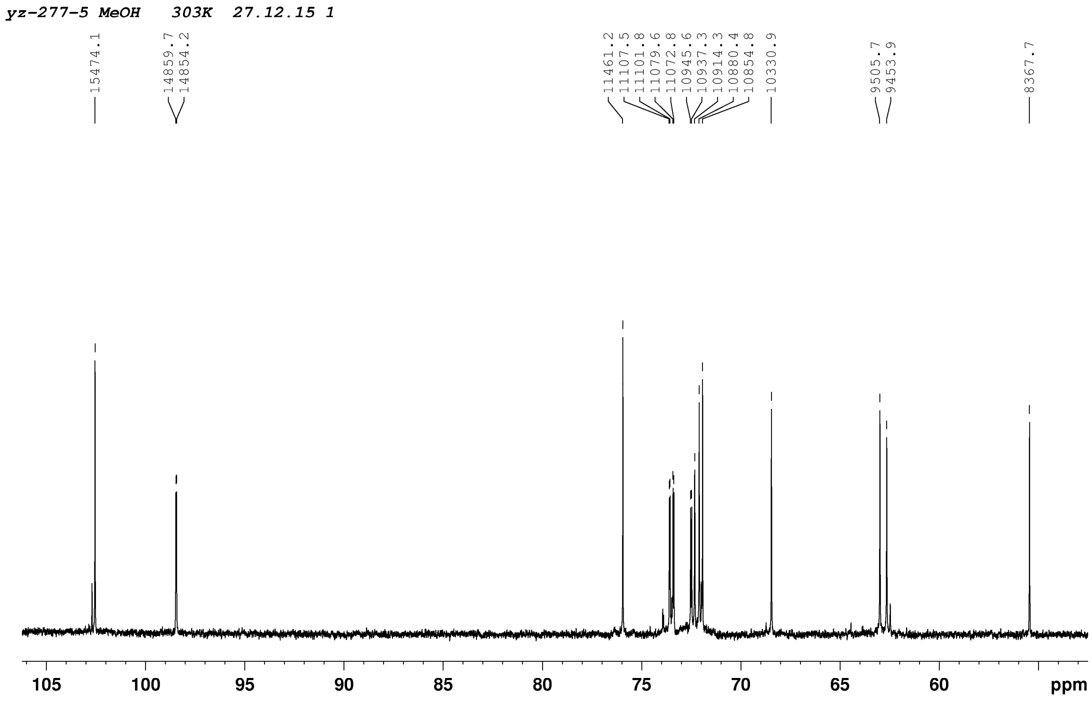

Figure S19. ^{13}C NMR spectra for compound **2**.

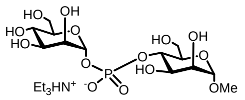

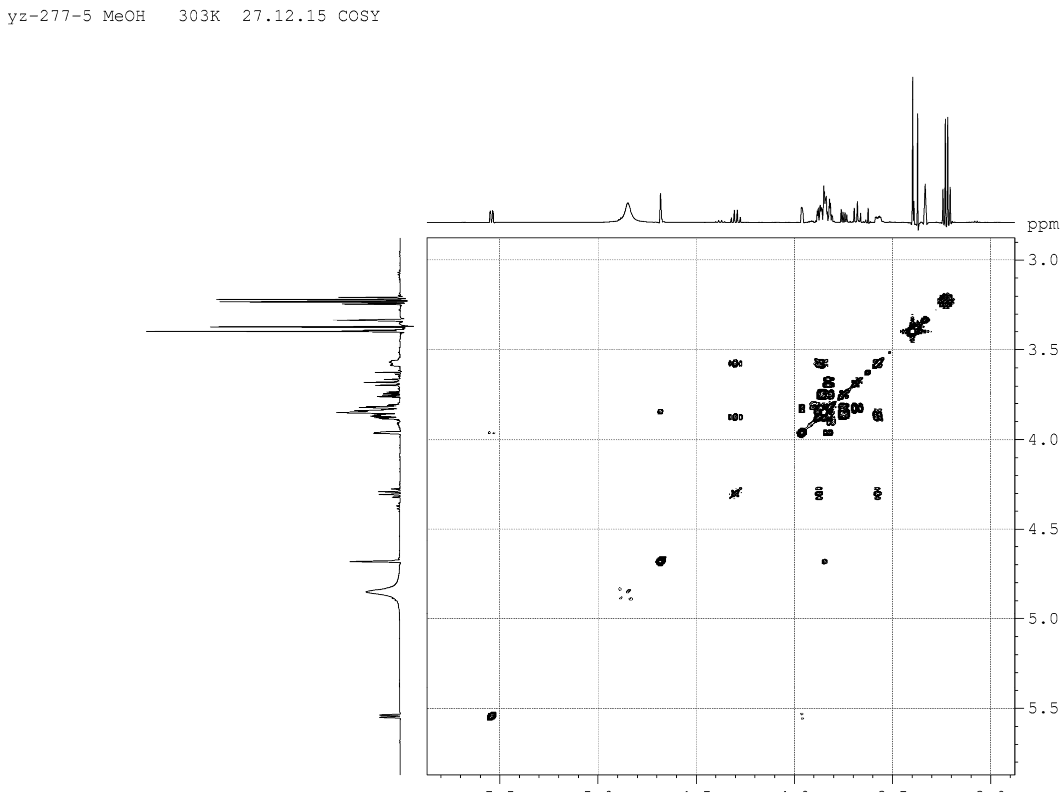

Figure S20. ^{1}H-^{1}H COSY spectrum for compound **2**.

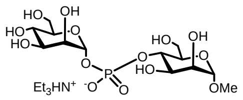

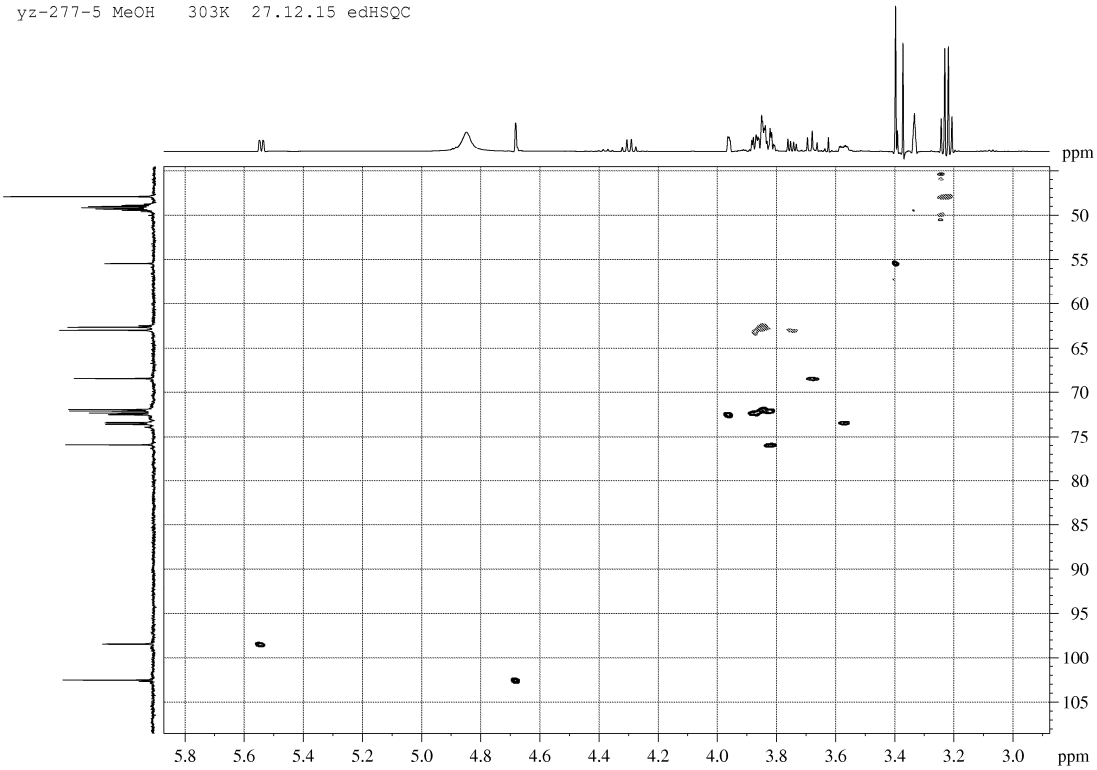

Figure S21. ^{1}H-^{13}C edHSQC spectrum for compound **2**.
